# Physiological strength of lipoic acid in copper intoxication

**DOI:** 10.1192/j.eurpsy.2022.578

**Published:** 2022-09-01

**Authors:** J. Jovanovic Mirkovic, G. Kocic, C. Alexopoulos, Z. Jurinjak

**Affiliations:** 1 The Academy of Applied Preschool Teaching and Health Studies, Biochemistry, Cuprija, Serbia; 2 Faculty of Medicine, Biochemistry, Nis, Serbia; 3 The Academy of Applied Preschool Teaching and Health Studies, Cuprija, Serbia, Medicine, Cuprija, Serbia

**Keywords:** Lipoic acid, copper, intoxication

## Abstract

**Introduction:**

The average concentration of copper in the air, e.g. in the USA it ranges from 5–20 ng/m3, in soil from 5–70 mg/kg, and the intake of copper from food is 1.0–1.3 mg/days for adults (0.014–0.019 mg/kg/day) (Barceloux, 1999). The effect of lipoic acid is reflected in the intensification of ATP synthesis, participates in the assimilation of lactic acid, activates the enzyme cycle of tricarboxylic acid, stimulates the growth of lactic acid bacteria by replacing acetate (acetate transfer factor), stimulates CoA synthesis (fatty acid utilization), prevents liver damage by various toxins, normalizes aldolase and transferase levels.

**Objectives:**

The aim of this study is to show the useful role of a supplement, lipoic acid, as an antioxidant in the prevention of oxidative stress.

**Methods:**

All procedures were performed after anesthesia of albino rats with ketal in accordance with the principles of sacrifice in laboratories. After medial laparotomy albino rates Wistar soy, a 10% homogenate of brain tissue was made in an appropriate medium and an analysis of acid and alkaline DNase activity was performed (Kocić i sar., 2004).

**Results:**

DNases are thought to be the main executors of apoptosis, responsible for internucleosomal DNA fragmentation, which is the breakdown of chromosomal DNA into oligonucleosome-sized fragments. Administration of lipoic acid has been shown to protect against oxidative stress caused by copper.

**Conclusions:**

Based on the results of this research, it can be concluded that lipoic acid is a powerful and powerful antioxidant.

**Disclosure:**

No significant relationships.

